# Aggregation-Induced Luminescent 3-Phenylpyrano[4,3-b]quinolizine Derivatives as Photosensitizers with Anti-Cancer Properties

**DOI:** 10.3390/molecules30071422

**Published:** 2025-03-23

**Authors:** Masayori Hagimori, Tatsusada Yoshida, Takuma Tsutsumi, Fumiko Hara, Shinya Takada, Yukiko Ogawa, Keitaro Tanaka

**Affiliations:** 1 Faculty of Pharmaceutical Sciences, Mukogawa Women’s University, 11-68 Koshien 9-Bancho, Nishinomiya 663-8179, Hyogo, Japan; fhara@mukogawa-u.ac.jp (F.H.); takada_shinya_x@mukogawa-u.ac.jp (S.T.); 2 Faculty of Pharmaceutical Sciences, Nagasaki International University, 2825-7 Huis Ten Bosch, Sasebo 859-3298, Nagasaki, Japan; tyoshida@niu.ac.jp (T.Y.); t.takuma0501@gmail.com (T.T.); yogawa@niu.ac.jp (Y.O.); 3 Saiki Central Hospital Pharmacy, 6-30 Tokiwa Higashi-machi, Saiki 876-0851, Oita, Japan; kei.tanaka1214@gmail.com

**Keywords:** 3-phenyl pyrano[4,3-b]quinolizine, fluorescence, AIEE, photodynamic therapy, photosensitizer

## Abstract

Photodynamic therapy (PDT) has garnered significant attention as an effective and safe method for cancer therapy, with ongoing efforts to develop new photosensitizers to enhance its efficacy. This study aimed to develop novel photosensitizers with aggregation-induced emission enhancement (AIEE) properties. A series of 3-phenyl pyrano[4,3-b]quinolizine compounds (**3**–**10**) were synthesized by reacting pyrones (**1a**–**e**) with 2-pyridylacetate (**2a**) or 2-pyridylacetonitrile (**2b**) and then evaluated for their potential as photosensitizers. Spectroscopic analyses revealed that all compounds emitted blue to green fluorescence in ethanol, with emission wavelengths ranging from 446 nm to 515 nm. Compounds **5** and **6**, lacking a substituent at position 5 of pyrano[4,3-b]quinolizine, exhibited AIEE behavior in aqueous solution. Furthermore, all compounds produced reactive oxygen species upon exposure to LED light. Notably, compounds **5** and **6** demonstrate high singlet oxygen (^1^O_2_) generation efficiency in water-rich solvents, where they tend to aggregate, contributing to their potential to destroy cancer cells. In vitro studies using human colon cancer cells (Colo205) demonstrated that **5** and **6** exhibited potent anti-tumor activity upon exposure to LED light. These findings suggest that compounds **5** and **6**, based on 3-phenyl pyrano[4,3-b]quinolizine, possessing AIEE properties, are potential new photosensitizers for PDT.

## 1. Introduction

Fluorescent materials are extensively utilized in the fields of life sciences and medicine owing to their high sensitivity and the ability to derive various information from their excitation and fluorescence spectra [1,2,3]. These materials are derived from small organic molecules, proteins, quantum dots, and others [4,5]. Small organic molecules are relatively easy to modify structurally and introduce substituents, enabling the optimization of emission intensity and wavelength according to the intended purpose, displaying characteristic fluorescence in dilute solutions [6,7,8]. However, at concentrations above a certain threshold, many fluorescent molecules tend to aggregate and quench, a phenomenon known as aggregation-caused quenching (ACQ) [9,10]. Consequently, the occurrence of ACQ in aqueous solutions raises concerns, as it may lead to unexpected outcomes in biological applications. In contrast, aggregation-induced emission enhancement (AIEE) dyes based on small organic molecules have been developed, and these dyes retain their fluorescence even after aggregation [11,12,13]. As a result, these AIEE dyes hold promise for diverse applications in various fields, including biology.

Cancer is one of the main causes of mortality worldwide, and conquering it is a desirable goal [14]. The primary treatments for cancer include surgery, chemotherapy, radiotherapy, and immunotherapy, either alone or in combination [15]. However, each treatment modality presents unique challenges, such as invasiveness, side effects, resistance, and lengthy treatment durations, necessitating the development of new cancer treatment modalities. Photodynamic therapy (PDT) is one of the treatment modalities that utilize the reaction induced by light irradiation on agents accumulated in the cancerous tissue [15]. PDT has garnered attention as a cancer-specific treatment in recent years. The agents used in PDT are photosensitizers that produce reactive oxygen species (ROS) upon exposure to light of particular wavelengths, facilitating the targeted destruction of cancer cells. Generally, ROS generated by photosensitizers fall into two categories: Type I, which yields superoxide anion (O_2_•^−^) and hydroxyl radical (•OH), and Type II, which produces singlet oxygen (^1^O_2_) [16,17]. In clinical settings, porphyrin derivatives such as talaporfin sodium and redaporfin are commonly utilized due to their efficient generation of ^1^O_2_ upon light exposure and proven efficacy in the treatment of various cancers, including breast cancer, melanoma, and head and neck cancer [18,19,20]. However, these photosensitizers have a tendency to aggregate in aqueous solutions, leading to reduced ROS production. Consequently, alternative agents are being developed to replace porphyrin derivatives. In this respect, AIEE dyes hold promise for stable ROS generation, even in an aggregated state. In recent years, attempts have been undertaken to develop photosensitizers based on this phenomenon [21,22,23].

In this study, to develop novel photosensitizers based on AIEE, we synthesized a series of 3-phenyl pyrano[4,3-b]quinolizine compounds (**3**–**10**). Pyrano[4,3-b]quinolizine is a tricyclic compound known for its fluorescence properties when electron-donating or electron-withdrawing groups are introduced. It has been utilized in fluorescent probes for targeting metal ions in biological and environmental samples [24,25]. Among these derivatives, compounds **5** and **6**, lacking a substituent at position 5 of pyrano[4,3-b]quinolizine, exhibited AIEE characteristics in aqueous media. This study reports the synthesis, photophysical properties, LED light-induced generation of ROS, and anti-tumor activity of the 3-phenyl pyrano[4,3-b]quinolizine compounds.

## 2. Results and Discussion

**Scheme 1:**
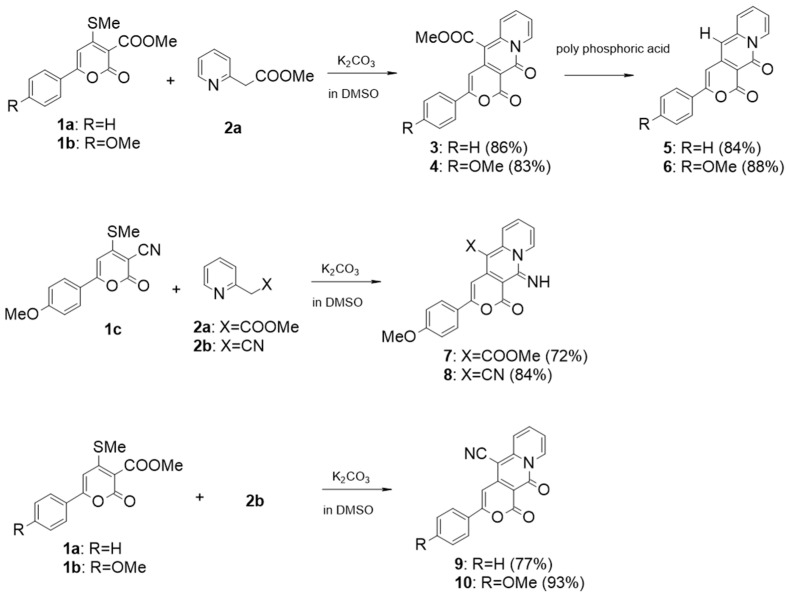
Syntheses of 3-phenylpyrano[4,3-b]quinolizine compounds (**3**–**10**).

As shown in Scheme 1, 3-phenylpyrano[4,3-b]quinolizine compounds (**3**–**10**) were synthesized by reacting pyrones (**1a**–**e**) with 2-pyridylacetate (**2a**) or 2-pyridylacetonitrile (**2b**) using a previously reported synthetic method [24]. In dimethyl sulfoxide (DMSO), **1a** and **2a** were mixed with potassium carbonate as the base. After 2 h, neutralization of the mixture with 10% hydrochloric acid yielded the dicarbonyl compound, methyl 1,11-dioxo-3-phenyl-1H,11H-pyrano[4,3-b]quinolizine-5-carboxylate (**3**), with an 86% yield. Similarly, the dicarbonyl compound **4** was obtained in an 83% yield from the reaction of **1b** and **2a**. Decarboxylated compounds (**5** and **6**) were obtained in good yield by treating the corresponding 3-phenylpyrano[4,3-b]quinolizines (**3** and **4**) with polyphosphoric acid (PPA), respectively. Imino carbonyl compounds (**7** and **8**) and dicarbonyl compounds with a cyano group (**9** and **10**) were synthesized with yields ranging from 72% to 93% using the same synthetic method as compound **3**. The molecular structures of **3**–**10** were verified through ^1^H- and ^13^C-NMR (Figures S1–S16 in the Supplementary Materials File).

Spectroscopic studies of compounds **3**–**10** in ultraviolet/visible (UV/Vis) and fluorescence were conducted in ethanol (EtOH) (Figures S17 and S18 in Supplementary Materials File). Table 1 lists the spectroscopic properties, including the maximum value of absorption wavelength (λ_max_), molar extinction coefficient (log ε), emission maximum (Em_max_), Stokes shift (SS), and quantum yield of fluorescence (Φ). The λ_max_ of dicarbonyl compounds **3**–**6**, **9**, and **10** ranged from 432 to 445 nm in EtOH. In contrast, the λ_max_ of imino carbonyl compounds **7** and **8** exhibited a hypsochromic shift at 366 and 368 nm, respectively. Additionally, the log ε of compounds **7** and **8** was lower than that of compounds **3**–**6**, **9**, and **10**, suggesting that they are influenced by the structural or electronic effects of the imino group at position 1 on pyrano[4,3-b]quinolizine. Regarding fluorescence, all compounds exhibited blue to green fluorescence ranging from 446 nm to 515 nm. Compounds **4**, **6**, and **10**, with a methoxy group at position 3 of pyrano[4,3-b]quinolizine, exhibited a bathochromic shift of 4–64 nm compared to compounds **3**, **5**, and **9** without a methoxy group. In contrast, de-esterification of compounds **3** and **4** led to a hypsochromic shift in Em_max_ for compounds **5** and **6**, accompanied by decreased Φ values (0.11 and 0.03, respectively). In compounds **4** and **7** with ester groups, the introduction of an imino group did not significantly alter fluorescence properties (Em_max_ and Φ values). However, in comparison to compounds **8** and **10** with cyano groups, the imino group shifted the fluorescence wavelength 44 nm towards longer wavelengths. Imino carbonyl compounds **7** and **8** exhibited large SS values exceeding 100 nm. Generally, the SS of organic fluorophores is in 10 s of nm, with values greater than 100 nm reported for lanthanide chelate complexes, nanoparticles, and proteins that exhibit fluorescence. A large SS is advantageous as it minimizes the effect of light scattering during fluorescence measurements [26].

**Figure 1:**
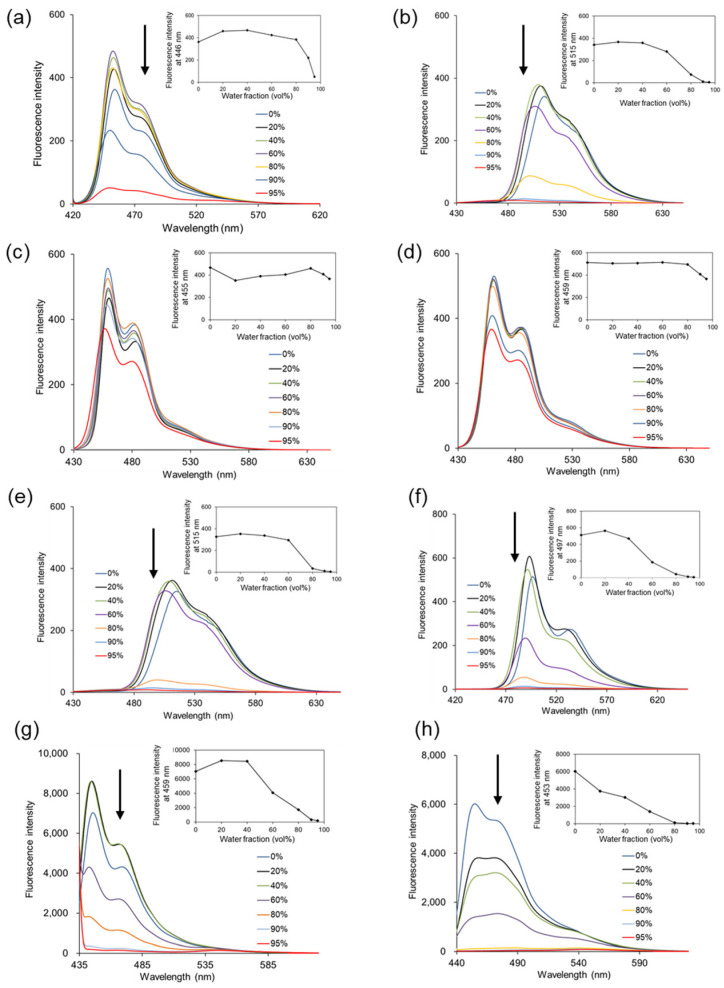
Fluorescence spectra of (**a**) **3** (10^−5^ M, λ_ex_ = 435 nm); (**b**) **4** (10^−5^ M, λ_ex_ = 438 nm); (**c**) **5** (10^−5^ M, λ_ex_ = 441 nm); (**d**) **6** (10^−5^ M, λ_ex_ = 445 nm); (**e**) **7** (10^−5^ M, λ_ex_ = 366 nm); (**f**) **8** (10^−5^ M, λ_ex_ = 368 nm) ); (**g**) **9** (10^−5^ M, λ_ex_ = 432 nm); (**h**) **10** (10^−5^ M, λ_ex_ = 434 nm) in H_2_O/ethanol mixture with different water fractions. Insets show the fluorescence intensity at each Em_max_. The arrows indicate the decrease in fluorescence with increasing water ratio.

The AIEE behaviors of 3-phenylpyrano[4,3-b]quinolizine compounds (**3**–**10**) were investigated in various EtOH/H_2_O (*v*/*v*) solvent mixtures (Figure 1). The fluorescence intensity of compounds **3**, **4**, and **7**–**10** decreased as the water ratio in the solvent increased. The fluorescence intensity at Em_max_ for each compound was plotted (insets in Figure 1) and was completely quenched when the solvent was nearly all water (95% water). This phenomenon is attributed to ACQ, resulting from aggregate formation with increasing water content in the measurement solvent. In contrast, compounds **5** and **6** exhibited consistent fluorescence intensity even in solvents with 90% or 95% water, showing no significant decrease with an increasing water ratio. A dynamic light scattering (DLS) study was conducted to examine whether compounds **3**–**10** aggregate as the water ratio in the solvent increases. All compounds increased in size as the water ratio increased, as shown in Table 2, suggesting that the fluorescence of compounds **5** and **6** in high-water-ratio solutions is due to AIEE. Furthermore, compounds **5** and **6** lack a substituent at position 5 of pyrano[4,3-b]quinolizine, indicating that this position of pyrano[4,3-b]quinolizine is primarily responsible for the AIEE effect.

**Figure 2:**
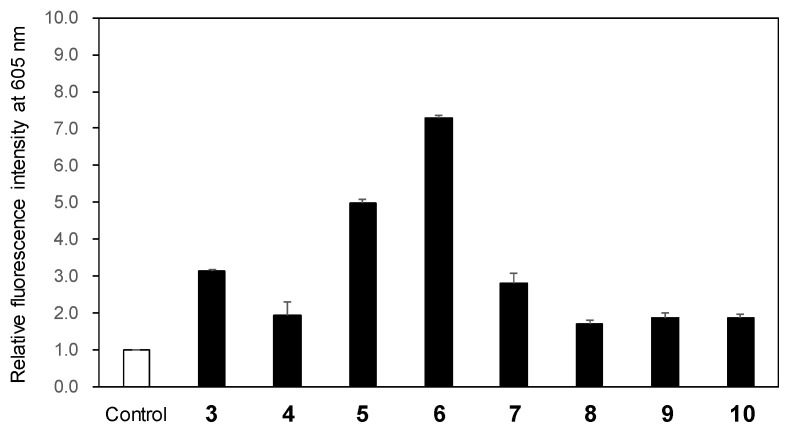
Relative ROS generation of acetonitrile solutions of **3**–**10** under 365 nm irradiation.

**Figure 3:**
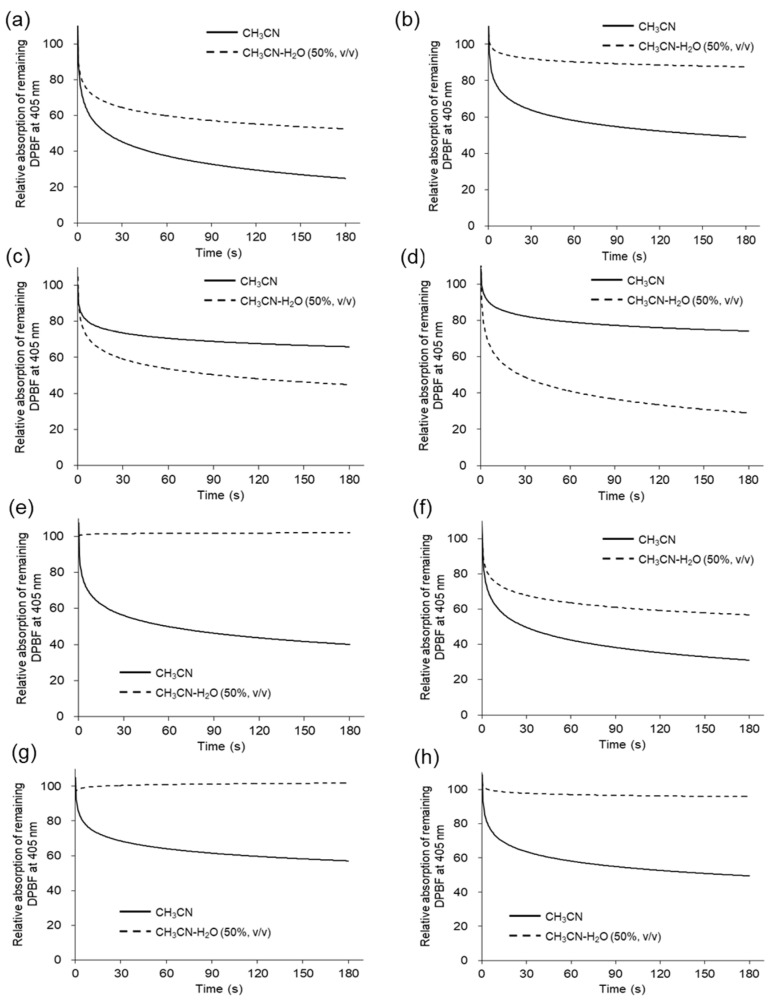
Change in absorbance of DPBF at 405 nm during the photodecomposition of DPBF by ^1^O_2_ in the presence of compounds **3**–**10** (2 × 10^−4^ M) upon irradiation at 365 nm. (**a**) **3**; (**b**) **4**; (**c**) **5**; (**d**) **6**; (**e**) **7**; (**f**) **8**; (**g**) **9**; (**h**) **10** in CH_3_CN (solid line) and CH_3_CN-H_2_O (50%, *v*/*v*) (dot line).

To evaluate the ROS generation following LED light exposure of 3-phenylpyrano[4,3-b]quinolizine compounds (**3**–**10**), we used the Amplite™ ROS Red total ROS detection sensor. Total ROS includes O_2_•^−^, •OH, hydrogen peroxide, ^1^O_2_, nitric oxide, and other related species. The fluorescence intensity at 605 nm of the sensor increases upon ROS production in the solution. After exposing the mixture of the sensor and each compound in acetonitrile to 365 nm LED light (1.7 W) for 2 min, the fluorescence spectrum excited at 520 nm was recorded. Compared to the fluorescence intensity of the sensor-only solution used as a control, an increase in fluorescence intensity was observed for all compounds, indicating ROS generation (Figure 2). Notably, compounds **5** and **6** exhibited 5.0- and 7.3-fold higher fluorescence intensity, respectively, relative to the control, signifying significant ROS generation. Given that the ^1^O_2_ is highly reactive and the major ROS responsible for cancer cell destruction in PDT, its generation was subsequently investigated [16,17]. 1,3-Diphenylisobenzofuran (DPBF) serves as a common ^1^O_2_ scavenger [27], with its absorption at 405 nm decreasing upon reaction with ^1^O_2_. Solutions of DPBF and each compound in acetonitrile were irradiated in a 96-well-plate with 365 nm LED light (1.7 W). The degradation of DPBF over LED light exposure time was tracked up to 180 sec via UV/Vis absorption. The degradation of DPBF in acetonitrile is illustrated by the solid line in Figure 3, indicating that all compounds generate ^1^O_2_. The observed rate constants and ^1^O_2_ quantum yield (Φ_Δ_) in acetonitrile are listed in Table 3. The Φ_Δ_ values in acetonitrile ranged from 0.53 to 0.91, with compounds **5** and **6** displaying relatively lower values of 0.53 and 0.67, respectively. The higher values of total ROS generation for compounds **5** and **6** in acetonitrile shown in Figure 2 may be due to the contribution of various ROS other than ^1^O_2_. Conversely, in acetonitrile/water 50:50 (*v*/*v*) (Figure 3, dotted line), the degradation of DPBF was more pronounced for compounds **5** and **6** than in acetonitrile alone. These findings suggest that compounds **5** and **6** lack a substituent at position 5 of pyrano[4,3-b]quinolizine, absorb light, and generate higher levels of singlet oxygen in environments with elevated water contents, where molecular aggregation is more probable.

**Figure 4:**
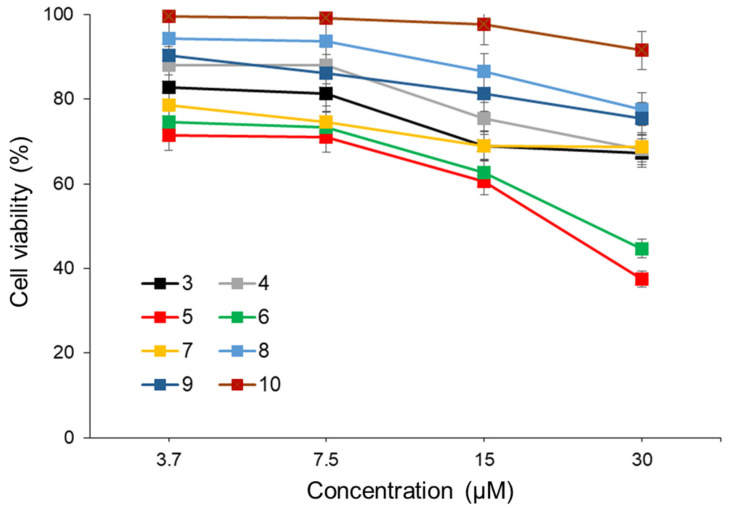
Anti-tumor activities of 3-phenyl pyrano[4,3-b]quinolizine compounds (**3**–**10**) against human colon cancer cells (Colo205) after 365 nm LED light irradiation.

The anti-tumor activity of compounds **3**–**10** as a photosensitizer in PDT was investigated using human colon cancer cells (Colo205). Colo205 cells were treated with compounds **3**–**10** at different concentrations for 4 h and then exposed to 365 nm LED light (482.4 mW) for 300 sec. Following an additional 48 h of incubation, the anti-tumor activity of **3**–**10** was assessed using the WST-8 assay. For all compounds, the cell viability in the non-irradiated LED group remained above 80%, and no significant effects were observed (Figure S19). As depicted in Figure 4, compounds **5** and **6** exhibited significant anti-tumor effects on Colo205 cells, with half maximal inhibitory concentrations (IC_50_) of 20.1 µM and 23.8 µM, respectively. The lack of anti-tumor effects in cells not exposed to LED light post-treatment with compounds **5** and **6** indicates that the anti-tumor activity was induced by ^1^O_2_ generation upon light exposure. These results suggest that compounds **5** and **6**, possessing AIEE properties, could serve as promising photosensitizers for PDT. However, further investigations, including assessments with other cancer cell lines, are warranted.

## 3. Materials and Methods

### 3.1. Reagents and Instruments

The materials and reactants utilized in this study were purchased from suppliers and utilized without purification if not specifically required. The following equipment were used in the identification and quantification of 3-phenyl pyrano[4,3-b]quinolizine compounds. A Yanako MP-500D (Kyoto, Japan) was used to determine the melting points. The JEOL JNM-EPC 400 (Tokyo, Japna) and JNM-ECA 500 (Tokyo, Japna) spectrometers were utilized to obtain the NMR spectrum. The JMS-700 mass spectrometer (JEOL, Tokyo, Japan) was used to acquire mass spectra (MS) and high-resolution mass spectrometry (HRMS). UV/Vis absorption data were acquired with a UV-2450 spectrometer (Shimadzu, Kyoto, Japan). The fluorescence data were acquired by an FP-8300 spectrometer (JASCO, Tokyo, Japan).

### 3.2. Synthetic Method of Methyl 1,11-dioxo-3-phenyl-1H,11H-pyrano[4,3-b]quinolizine-5-carboxylate (***3***) [24]

To a mixture of 0.55 g of pyrone **1a** (2.0 mmol) and 0.45 g of methyl 2-pyridylacetate **2a** (3.0 mmol) in 20 mL of DMSO, 0.67 g of potassium carbonate (5.0 mmol) was mixed and stirred at room temperature. After 2 h of stirring, the mixture was reacted for a further 20 min at 50–60 °C. Subsequently, 200 mL of ice water was added into the mixture, leading to the generation of a precipitate that was collected through filtration and rinsed with water. Recrystallization using DMF gave **3** (0.59 g, 1.7 mmol) of orange needles in 86% yield. Melting point: 239–241 °C. ^1^H NMR (400 MHz, CDCl_3_) δ: 9.39 (d, *J* = 5.8 Hz, 1H), 8.09 (d, *J* = 8.8 Hz, 1H), 7.91 (m, 2H), 7.74 (m, 1H), 7.48 (m, 3H), 7.24 (m, 1H), 7.21 (s, 1H) 4.08 (s, 3H). ^13^C NMR (100 MHz, CDCl_3_) δ: 175.1, 166.3, 159.6, 158.6, 154.9, 146.3, 145.7, 136.5, 131.4, 131.3, 130.1, 128.9, 126.2, 123.7, 116.2, 101.2, 97.9, 96.7, 52.6. MS spectrum (ESI, *m*/*z*): 348 (M^+^+1), 347 (M^+^). HRMS (ESI, *m*/*z*): Calcd. for C_20_H_13_NO_5_, 347.0794 (M^+^); found, 347.0793.

### 3.3. Synthetic Method of Methyl 1,11-dihydro-3-(4-methoxy)phenyl-1,11-dioxoyrano[4,3-b]quinolizine-5-carboxylate (***4***) [24]

The desired compound **4** (0.31 g, 0.83 mmol) was synthesized with an 83% yield from 0.31 g of **1b** (1.0 mmol) and 0.22 g of 2-pyridylacetonitrile **2b** (1.5 mmol) following a procedure similar to the synthetic method of **3**. The sample for analysis was recrystallized from DMF to obtain yellow needles, with a melting point of 256–260 °C. ^1^H NMR (400 MHz, CDCl_3_) δ: 9.39 (d, *J* = 3.0 Hz, 1H), 8.06 (d, *J* = 8.0 Hz, 1H), 7.86 (m, 1H), 7.70 (m, 1H), 7.17 (m, 1H), 7.13 (s, 1H), 7.00 (m, 2H), 4.07 (s, 3H), 3.88 (s, 3H). ^13^C NMR (100 MHz, CDCl_3_) δ: 166.4, 162.2, 159.7, 158.7, 154.9, 146.6, 145.7, 136.3, 130.1, 128.4, 128.0, 123.6, 115.9, 114.3, 100.9, 96.3, 55.6, 52.6. MS spectrum (ESI, *m*/*z*): 378 (M^+^+1), 377 (M^+^). Anal. Calcd. for C_21_H_15_NO_6_ 377.0899: C 66.84 H, 4.01; N 3.71. Found: C, 66.79; H, 4.12; N, 3.59.

### 3.4. Synthetic method of 1,11-dihydro-3-phenylpyrano[4,3-b]quinolizine-1,11-dione (***5***) [24]

A mixture of 1.74 g of **3** (5.0 mmol) and 30 g of PPA was heated with stirring at 100 °C for 1 h. Upon adding ice water to the mixture, the resulting residue was collected through filtration and rinsed with water. Recrystallization using DMF gave **5** (1.21 g, 4.2 mmol) of orange needles in 84% yield. Melting point: 256–257 °C. ^1^H NMR (400 MHz, CDCl_3_) δ: 9.22 (d, *J* = 6.8 Hz, 1H), 7.94 (d, *J* = 7.8 Hz, 1H), 7.89 (m, 2H), 7.54 (m, 1H), 7.46 (m, 3H), 7.02 (m, 1H), 6.70 (s, 1H), 6.47 (s, 1H). ^13^C NMR (100 MHz, CDCl_3_) δ: 158.9, 155.8, 149.1, 146.8, 146.1, 134.3, 131.3, 130.9, 129.5, 129.4, 128.8, 126.9, 125.9, 125.1, 122.5, 115.2, 99.7, 98.4. MS spectrum (ESI, *m*/*z*): 290 (M^+^+1), 289 (M^+^). HRMS (ESI, *m*/*z*): Calcd. for C_18_H_11_NO_3_, 289.0739 (M^+^); found, 289.0743.

### 3.5. Synthetic Method of 1,11-dihydro-3-(4-methoxy-phenyl)-pyrano[4,3-b]quinolizine-1,11-dione (***6***) [24]

The desired compound **6** (0.28 g, 0.88 mmol) was synthesized with an 88% yield from 0.38 g of **4** (1.0 mmol) and 30 g of PPA following a procedure similar to the synthesis of **5**. The sample for analysis was recrystallized from DMF to obtain yellow needles, with a melting point of 214–216 °C. ^1^H NMR (400 MHz, DMSO-*d*_6_) δ: 9.03 (d, *J* = 7.3 Hz, 1H), 7.98 (d, *J* = 7.3 Hz, 1H), 7.81 (d, *J* = 8.3 Hz, 1), 7.75 (m, 1H), 7.28 (m, 1H), 7.26 (d, *J* = 8.3 Hz, 2H), 7.07 (s, 1H), 6.69 (s, 1H), 3.83 (s, 3H). ^13^C NMR (100 MHz, DMSO-*d*_6_) δ: 161.4, 157.9, 156.7, 154.9, 146.0, 135.4, 127.0, 125.6, 120.5, 116.3, 99.9, 95.2, 55.5. MS spectrum (ESI, *m*/*z*): 320 (M^+^+1), 319 (M^+^). HRMS (ESI, *m*/*z*): Calcd. for C_19_H_13_NO_4_, 319.0845 (M^+^); found, 319.0851.

### 3.6. Synthetic Method of Methyl 1,11-dihydro-11-imino-3-(4-methoxyphenyl)-1-oxopyrano[4,3-b]quinolizine-5 carboxylate (***7***) [24]

To a mixture of 0.55 g of **1c** (2.0 mmol) and 0.30 g of **2a** (2.0 mmol) in 10 mL of DMSO, 0.12 g of potassium carbonate (0.9 mmol) was added and stirred at room temperature. After 2 h of stirring, the mixture was stirred for another 30 min at 50–60 °C. Subsequently, 200 mL of ice water was poured into the reaction mixture, leading to the formation of a precipitate that was collected through filtration and rinsed with water. Recrystallization using DMF gave 0.54 g of **7** (1.4 mmol) of orange needles in 72% yield. Melting point: 244–245 °C. ^1^H NMR (400 MHz, CDCl_3_) δ: 10.38 (br s, 1H), 9.72 (d, *J* = 6.6 Hz, 1H), 8.05 (dd, *J* = 1.5, 9.1 Hz, 1H), 7.85 (d, *J* = 9.1 Hz, 2H), 7.62 (ddd, *J* = 1.5, 6.6, 9.1 Hz, 1H), 7.26 (s, 1H), 7.03 (dd, *J* = 6.6, 6.6 Hz, 1H), 6.99 (d, *J* = 9.1 Hz, 2H), 4.00 (s, 3H), 3.90 (s, 3H). ^13^C NMR (100 MHz, CDCl_3_) δ: 166.8, 162.2, 161.9, 158.4, 152.9, 146.9, 144.8, 136.6, 131.2, 127.7, 123.9, 123.3, 115.0, 114.3, 97.4, 96.7, 95.0, 55.5, 52.1. MS spectrum (ESI, *m*/*z*): 377 (M^+^+1), 376 (M^+^). Anal. Calcd. for C_20_H_14_N_2_O_4_ 376.11: C, 67.02; H, 4.28; N, 7.44. Found: C, 66.83; H, 4.20; N, 7.32.

### 3.7. Synthetic Method of 1,11-dihydro-1-imino-3-(4-methoxyphenyl)-1-oxopyrano[4,3-b]quinolizine-5-carbonitrile (***8***) [24]

This desired compound **8** (0.58 g, 0.17 mmol) was synthesized with an 84% yield from 0.55 g of **1c** (2.0 mmol) and 0.35 g of **2b** (3.0 mmol) following a procedure similar to the synthesis of **7**. The sample for analysis was recrystallized from DMF to obtain yellow needles, with a melting point of 280–282 °C. ^1^H NMR (400 MHz, CDCl_3_) δ: 10.48 (s, 1H), 9.13 (d, *J* = 7.2 Hz, 1H), 7.91 (d, *J* = 9.0 Hz, 2H), 7.82 (m, 1H), 7.16 (m, 1H), 7.01 (d, *J* = 9.0 Hz, 1H), 6.91 (s, 1H), 3.90 (s, 3H). ^13^C NMR (100 MHz, CDCl_3_) δ: 162.6, 161.1, 160.8, 151.9, 148.7, 146.5, 138.7, 131.7, 128.1, 123.0, 122.8, 116.5, 116.0, 114.5, 96.6, 95.0, 55.5. MS spectrum (ESI, *m*/*z*): 344 (M^+^+1), 343 (M^+^). Anal. Calcd. for C_20_H_13_N_3_O_3_ 343.34: C, 69.96; H, 3.82; N, 12.24. Found: C, 69.96; H, 3.78; N, 12.20.

### 3.8. Synthetic Method of 1,11-dioxo-3-phenyl-1H,11H-pyrano[4,3-b]quinolizine-5-carbonitrile (***9***)

This desired compound **9** (0.072 g, 0.23 mmol) was synthesized with a 77% yield from 0.10 g of **1a** (0.30 mmol) and 0.035 g of **2b** (0.30 mmol) following a procedure similar to the synthesis of **7**. The sample for analysis was recrystallized from DMF to obtain yellow needles, with a melting point of 344–345 °C. ^1^H NMR (500 MHz, CDCl_3_) δ: 9.42 (d, *J* = 7.5 Hz, 1H), 8.05 (d, *J* = 9.0 Hz, 1H), 8.01 (d, *J* = 8.0 Hz, 2H), 7.97 (m, 1H), 7.54 (m, 3H), 7.35 (m, 1H), 7.12 (s, 1H). ^13^C NMR (125 MHz, CDCl_3_) δ: 162.2, 157.4, 154.1, 149.5, 148.0, 138.9, 132.2, 130.9, 130.6, 129.1, 126.6, 123.3, 117.2, 115.3, 97.4, 96.7, 80.6. MS spectrum (ESI, *m*/*z*): 314 [M^+^]. HRMS (ESI, *m*/*z*): Calcd. for C_19_H_10_N_2_O_3_ [M^+^]: 314.0691. Found: 314.0691.

### 3.9. Synthetic Method of 3-(4-methoxyphenyl)-1,11-dioxo-1H,11H-pyrano[4,3-b]quinolizine-5-carbonitrile (***10***)

This desired compound **10** (0.32 g, 0.93 mmol) was synthesized with a 93% yield from 0.34 g of **1b** (1.0 mmol) and 0.12 g of **2b** (1.0 mmol) following a procedure similar to the synthesis of **7**. The sample for analysis was recrystallized from DMF to obtain yellow needles, with a melting point of 323–324 °C. ^1^H NMR (500 MHz, CDCl_3_) δ: 9.38 (d, *J* = 7.0 Hz, 1H), 8.02 (d, *J* = 9.0 Hz, 1H), 7.97 (m, 3H), 7.31 (dd, *J* = 6.5, 7.0 Hz, 1H), 7.03 (s, 1H), 7.00 (d, *J* = 9.5 Hz, 2H), 3.90 (s, 3H). ^13^C NMR (125 MHz, CDCl_3_) δ: 163.0, 162.3, 157.6, 154.1, 149.7, 148.1, 138.7, 130.8, 128.5, 123.2, 122.9, 116.9, 115.4, 114.6, 96.2, 95.7, 80.3, 55.6. MS spectrum (ESI, *m*/*z*): 344 [M^+^]. HRMS (ESI, *m*/*z*): Calcd. for C_20_H_12_N_2_O_4_ [M^+^]: 344.1797. Found: 344.0799.

### 3.10. Experimental Procedure of Spectral Measurements

UV/Vis absorption: Each compound was dissolved in DMSO to obtain a stock solution of 10^−2^ mol/L. Upon measuring, solutions of compounds (10^−4^ mol/L) were formulated utilizing a measurement solvent before the UV/Vis absorption analysis. After placing the solution in a cuvette and calibrating the UV/Vis spectrometer, the UV/Vis absorption spectra were acquired by scanning from 250 nm to 500 nm. Fluorescence: The slit widths for excitation and emission were set to 5 nm band pass, and the scan speed was set to 1000 nm/min. Each compound was dissolved in DMSO (FUJIFILM Wako Chemicals, Osaka, Japan) to obtain a stock solution of 10^−2^ mol/L. Upon measuring, a solution of compounds (10^−5^ mol/L) was formulated utilizing a measurement solvent before the fluorescence analysis. The solution was placed in a quartz cell, set in a fluorescence spectrometer, and the excitation wavelength of the evaluated compound was measured by examining the emission wavelength. Similarly, the emission wavelength was determined by determining the excitation wavelength of the evaluated compounds. By repeating this procedure, the exact excitation and emission wavelengths for each compound were obtained. The quantum yield of fluorescence (Φ) was determined with rhodamine B as a reference (Φ = 0.7 in EtOH).

### 3.11. Evaluation of the Aggregation Behavior

The aggregation behavior of each compound was evaluated using the dynamic light scattering (DLS) analysis with the Zetasizer Pro (Malvern Panalytical Ltd., Malvern, UK). Samples were diluted in EtOH and water solutions of different ratios, and the particle size was measured. The average of three measurements for each sample was utilized.

### 3.12. Total ROS Generation Activity Assay

The total ROS generation activity after light exposure to compounds (**3**–**10**) was verified using the Cell Meter™ Fluorimetric Intracellular Total ROS Activity Assay Kit Red Fluorescence (AAT Bioquest, Inc., Sunnyvale, CA, USA). The evaluated compounds, initially dissolved in DMSO at 10 mM, subsequently diluted in acetonitrile to an end-point concentration of 200 μM, had 1 μL of Amplite™ ROS RED working solution added. The solution was exposed to LED light (365 nm, 1.7 W, EL-FSE-02-A, Ebisudenshi Co., Ltd., Osaka, Japan) for 120 sec. The control consisted of an acetonitrile solution without any compound. Fluorescence spectra excited at 520 nm were recorded using an FP-8300 spectrometer (JASCO, Tokyo, Japan).

### 3.13. ^1^O_2_ Generation Activity Assay

DPBF was used to confirm the ^1^O_2_ generation activity. In a 96-well plate, 10 μL of DPBF in DMSO (10 mM) was blended with 2 μL of compounds **3**–**10** in DMSO (10 mM). Subsequently, this solution was made up to 100 μL with acetonitrile or a 50:50 (*v*/*v*) mixture of acetonitrile and water. After exposure to light (365 nm, 1.7 W, EL-FSE-02-A, Ebisudenshi Co., Ltd., Osaka, Japan) for up to 180 sec, the absorbance data at 405 nm were obtained using a microplate reader (Multiskan FC, Thermo Fisher Scientific, Waltham, MA, USA). The ^1^O_2_ quantum yield (Φ_Δ_) was calculated as the initial observed rate constant (*k*) of DPBF using the following equation:

Φ_Δx_ = Φ_Δ SP_ · (*k*_x_/*k*_SP_) · (F_SP_/F_x_)

where SP represents benzophenone, which is utilized as a standard photosensitizer; Φ_Δ SP_ denotes the Φ_Δ_ value of benzophenone (0.39 in acetonitrile); and F signifies the absorption cofactor, expressed as F = 1 − 10^OD^, where OD represents the optical density at the irradiated wavelength.

### 3.14. Investigation of In Vitro Anti-Tumor Activity

Human colorectal adenocarcinoma Colo205 cells were cultured in RPMI 1640 media with 10% (*v*/*v*) heat-inactivated fetal calf serum, penicillin (100 IU/mL), and streptomycin (100 μg/mL) at 37 °C in a 95% air and 5% CO_2_ atmosphere. In 96-well plates, Colo205 cells were seeded at a density of 1  × 10^4^ cells/well and cultured for 24 h. Compounds **3**–**10** (0–30 μM) were then added to the culture medium. After 4 h, the plates were exposed to 365 nm LED light irradiation. Following 48 h of incubation, cytotoxicity and cell viability/growth were assessed using 0.5% (*w*/*v*) trypan blue exclusion and 10 μL WST-8 assays. The reductions in the fraction of viable cells were determined using the GloMax Multi Detection System (Promega Corp., Madison, WI, USA) to measure absorbance at 450 nm (reference at 600 nm).

## 4. Conclusions

To develop new photosensitizers for PDT in cancer treatment, we synthesized 3-phenyl pyrano[4,3-b]quinolizine compounds (**3**–**10**) with moderate-to-good yields by reacting pyrones (**1a**–**e**) with 2-pyridylacetate (**2a**) or 2-pyridylacetonitrile (**2b**). Photophysical analysis showed that all compounds emitted blue-to-green fluorescence with wavelengths ranging from 446 nm to 515 nm in ethanol. Specifically, only compounds **5** and **6**, lacking a substituent at position 5 of the pyrano[4,3-b]quinolizine, maintained strong fluorescence as the water content in the solvent increased, indicating AIEE properties. In terms of ROS generation, all compounds produced ROS, particularly ^1^O_2_, upon light exposure. Interestingly, compounds **5** and **6**, which exhibited AIEE properties, demonstrated significantly increased ^1^O_2_ generation in the aggregated state. Furthermore, in assays using Colo205 cells, compounds **5** and **6** showed potent anti-tumor activity following LED light exposure. These findings suggest that 3-phenyl pyrano[4,3-b]quinolizine-based compounds **5** and **6** have the potential to serve as innovative photosensitizers for PDT in cancer treatment. Further evaluations across a wider range of cancer cell lines are planned to validate their efficacy.

## Figures and Tables

**Table 1:** Fluorescence data for **3**–**10** in EtOH.

Compounds	λ_max_ (nm)	log ε	Em_max_ (nm)	SS ^[a]^	Φ_FL_^[b]^
**3**	435	4.15	451	16	0.18
**4**	438	3.97	515	77	0.21
**5**	441	3.97	455	14	0.11
**6**	445	4.36	459	14	0.03
**7**	366	3.16	515	149	0.20
**8**	368	3.88	497	129	0.21
**9**	432	4.01	446	14	0.21
**10**	434	4.23	453	19	0.28

^[a]^ Each emission was measured using excitation wavelengths. ^[b]^ Fluorescence quantum yields were determined by using rhodamine B as a reference.

**Table 2:** Particle size of **3**–**10** in EtOH–water mixture.

Water Fraction (vol%)					
	0	30	50	80	95
**3**	2.0 ± 1.2	1.1 ± 0.6	57.0 ± 97.0	543.5 ± 377.9	460.3 ± 235.5
**4**	25.7 ± 44.5	72.5 ± 121.2	263.6 ± 37.4	306.4 ± 245.5	383.9 ± 354.4
**5**	0.7 ± 0.0	0.7 ± 0.1	24.1 ± 40.1	29.8 ± 46.2	265.3 ± 45.5
**6**	0.0 ± 0.0	0.9 ± 0.2	235.6 ± 0.1	296.9 ± 257.7	248.2 ± 73.6
**7**	18.8 ± 13.9	8.4 ± 12.3	227.1 ± 38.6	460.0 ± 330.9	76.4 ± 23.0
**8**	22.2 ± 38.5	24.8 ± 37.2	24.4 ± 36.7	375.1 ± 525.6	291.4 ± 61.4
**9**	17.6 ± 30.4	426.4 ± 344.4	508.5 ± 223.7	585.2 ± 19.1	589.4 ± 195.5
**10**	22.6 ± 36.1	354.9 ± 95.5	802.4 ± 222.0	990.6 ± 176.1	411.9 ± 179.2

Average ± S.D. (nm).

**Table 3:** Observed rate constant and singlet oxygen quantum yields of **3**–**10** in acetonitrile.

Compounds	Observed Rate Constant (*k*) [min^−1^]	Φ_Δ_^[a]^
**3**	1.01	0.91
**4**	0.95	0.85
**5**	0.59	0.53
**6**	0.75	0.67
**7**	0.84	0.75
**8**	0.92	0.83
**9**	0.82	0.73
**10**	0.81	0.72

^[a]^ Singlet oxygen quantum yields were determined by using benzofuran as a reference.

## Data Availability

The data presented in this study are available in the article.

## Supplementary Materials

The following supporting information can be downloaded at: https://www.mdpi.com/article/10.3390/molecules30071422/s1, Figure S1: ^1^H NMR spectrum (400 MHz, CDCl_3_) of **3**; Figure S2: ^13^C NMR spectrum (100 MHz, CDCl_3_) of **3**; Figure S3: ^1^H NMR spectrum (400 MHz, CDCl_3_) of **4**; Figure S4: ^13^C NMR spectrum (100 MHz, CDCl_3_) of **4**; Figure S5: ^1^H NMR spectrum (400 MHz, CDCl_3_) of **5**; Figure S6: ^13^C NMR spectrum (100 MHz, CDCl_3_) of **5**; Figure S7: ^1^H NMR spectrum (400 MHz, DMSO-*d*_6_) of **6**; Figure S8: ^13^C NMR spectrum (100 MHz, DMSO-*d*_6_) of **6**; Figure S9: ^1^H NMR spectrum (400 MHz, CDCl_3_) of **7**; Figure S10: ^13^C NMR spectrum (100 MHz, CDCl_3_) of **7**; Figure S11: ^1^H NMR spectrum (400 MHz, CDCl_3_) of **8**; Figure S12: ^13^C NMR spectrum (100 MHz, CDCl_3_) of **8**; Figure S13: ^1^H NMR spectrum (500 MHz, CDCl_3_) of **9**; Figure S14: ^13^C NMR spectrum of (125 MHz, CDCl_3_) **9**; Figure S15: ^1^H NMR spectrum (500 MHz, CDCl_3_) of **10**; Figure S16: ^13^C NMR spectrum (125 MHz, CDCl_3_) of **10**; Figure S17: UV/Vis spectrum of **3**–**10** in EtOH; Figure S18: Fluorescence spectrum of **3**–**10** in EtOH. Figure S19: Anti-tumor activities of 3-phenyl pyrano[4,3-b]quinolizine compounds (**3**–**10**) against human colon cancer cells (Colo205) without 365 nm LED light irradiation.
