# Supplementary material for: Aggregation-Induced Luminescent 3-Phenylpyrano[4,3-b]quinolizine Derivatives as Photosensitizers with Anti-Cancer Properties
Source: Molecules. 2025 Mar 23;30(7):1422. doi: 10.3390/molecules30071422 (PMC11990754; doi:10.3390/molecules30071422)
Supplement: Supplementary file 1 [file molecules-30-01422-s001.zip › molecules-3529693-supplementary.pdf]

Supplementary Materials

Aggregation-induced luminescent 3-phenyl pyrano[4,3-b]quinolizine derivatives as photosensitizers with anti-cancer properties

Masayori Hagimori [1,*], Tatsusada Yoshida [2], Takuma Tsutsumi [2], Fumiko Hara [1], Shinya Takada [1], Yukiko Ogawa [2], Keitaro Tanaka [3]

[1]Faculty of Pharmaceutical Sciences, Mukogawa Women's University, 11-68 Koshien 9-Bancho, Nishinomiya City 663-8179, Hyogo, Japan

[2]Faculty of Pharmaceutical Sciences, Nagasaki International University, 2825-7, Huis Ten Bosch, Sasebo City 859-3298, Nagasaki, Japan

[3]Saiki Central Hospital Pharmacy, 6-30 Tokiwa Higashi-machi, Saiki City, 876-0851, Oita, Japan

Contents

1. Figure S1. ^{1}H NMR spectrum (400 MHz, $CDCl_3$) of **3**

2. Figure S2. ^{13}C NMR spectrum (100 MHz, $CDCl_3$) of **3**

3. Figure S3. ^{1}H NMR spectrum (400 MHz, $CDCl_3$) of **4**

4. Figure S4. ^{13}C NMR spectrum (100 MHz, $CDCl_3$) of **4**

5. Figure S5. ^{1}H NMR spectrum (400 MHz, $CDCl_3$) of **5**

6. Figure S6. ^{13}C NMR spectrum (100 MHz, $CDCl_3$) of **5**

7. Figure S7. ^{1}H NMR spectrum (400 MHz, DMSO-d_6) of **6**

8. Figure S8. ^{13}C NMR spectrum (100 MHz, DMSO-d_6) of **6**

9. Figure S9. ^{1}H NMR spectrum (400 MHz, $CDCl_3$) of **7**

10. Figure S10. ^{13}C NMR spectrum (100 MHz, $CDCl_3$) of **7**

11. Figure S11. ^{1}H NMR spectrum (400 MHz, $CDCl_3$) of **8**

12. Figure S12. ^{13}C NMR spectrum (100 MHz, $CDCl_3$) of **8**

13. Figure S13. ^{1}H NMR spectrum (500 MHz, $CDCl_3$) of **9**

14. Figure S14. ^{13}C NMR spectrum of (125 MHz, $CDCl_3$) **9**

15. Figure S15. ^{1}H NMR spectrum (500 MHz, $CDCl_3$) of **10**

16. Figure S16. ^{13}C NMR spectrum (125 MHz, $CDCl_3$) of **10**

17. Figure S17. UV-Vis spectrum of **3-10** in EtOH

18. Figure S18. Fluorescence spectrum of **3-10** in EtOH

19. Figure S19. Anti-tumor activities of 3-phenyl pyrano[4,3-b]quinolizine compounds (**3-10**) against human colon cancer cells (Colo205) without 365 nm LED light irradiation.

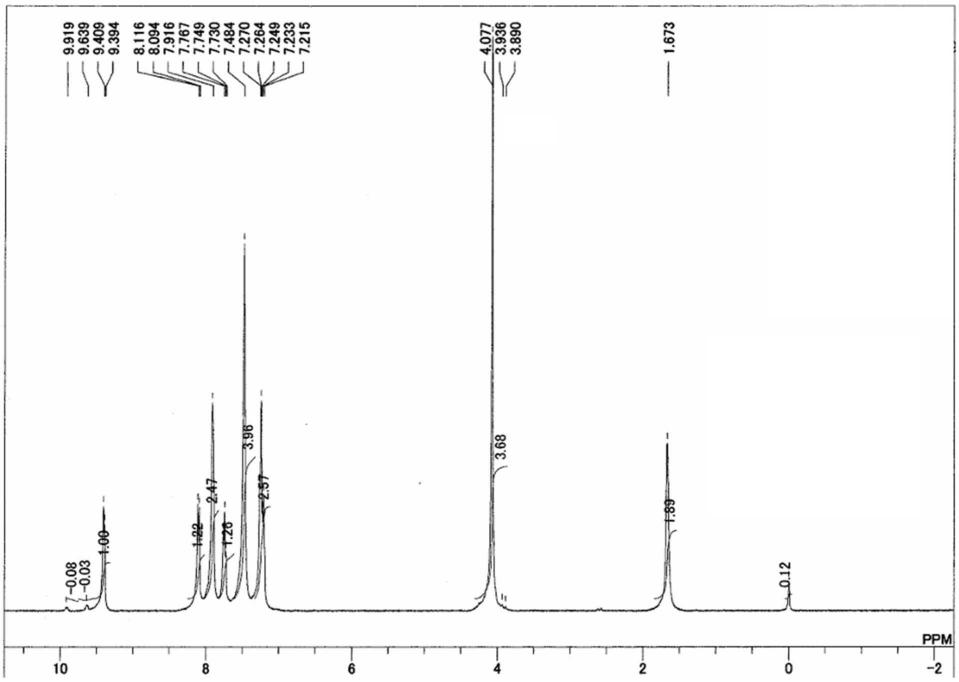

Figure S1. ^{1}H NMR spectrum (400 MHz, $CDCl_3$) of **3**.

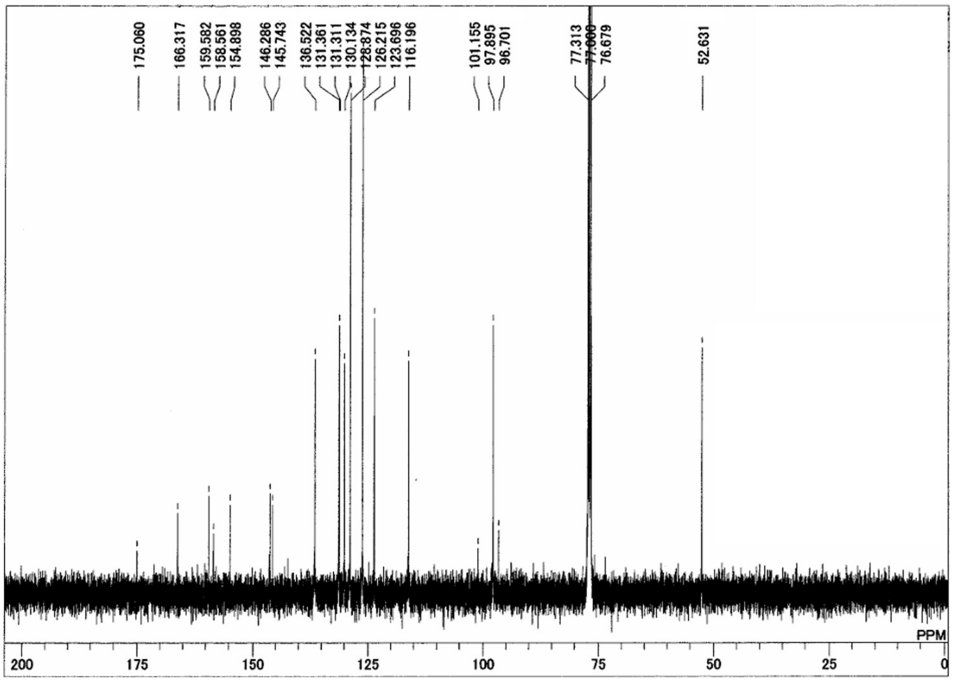

Figure S2. ^{13}C NMR spectrum (100 MHz, $CDCl_3$) of **3**.

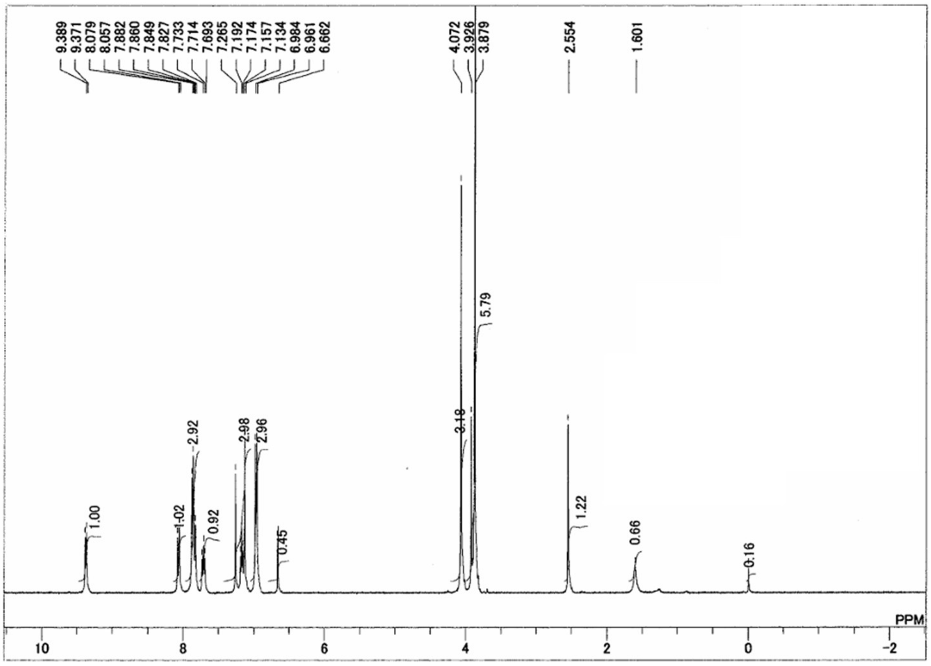

Figure S3. ^{1}H NMR spectrum (400 MHz, $CDCl_3$) of **4**.

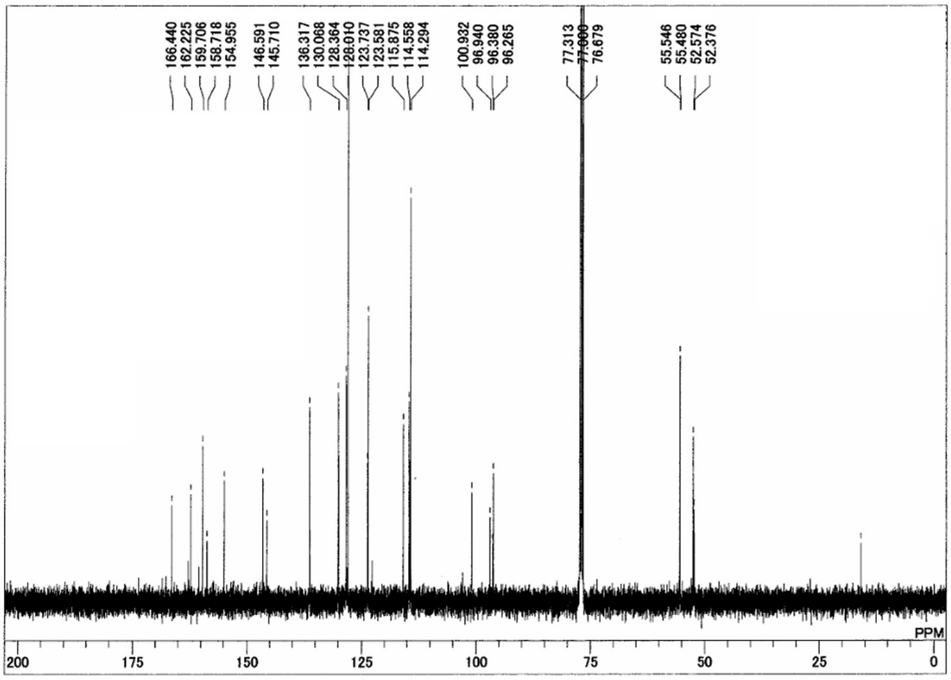

Figure S4. ^{13}C NMR spectrum (100 MHz, $CDCl_3$) of **4**.

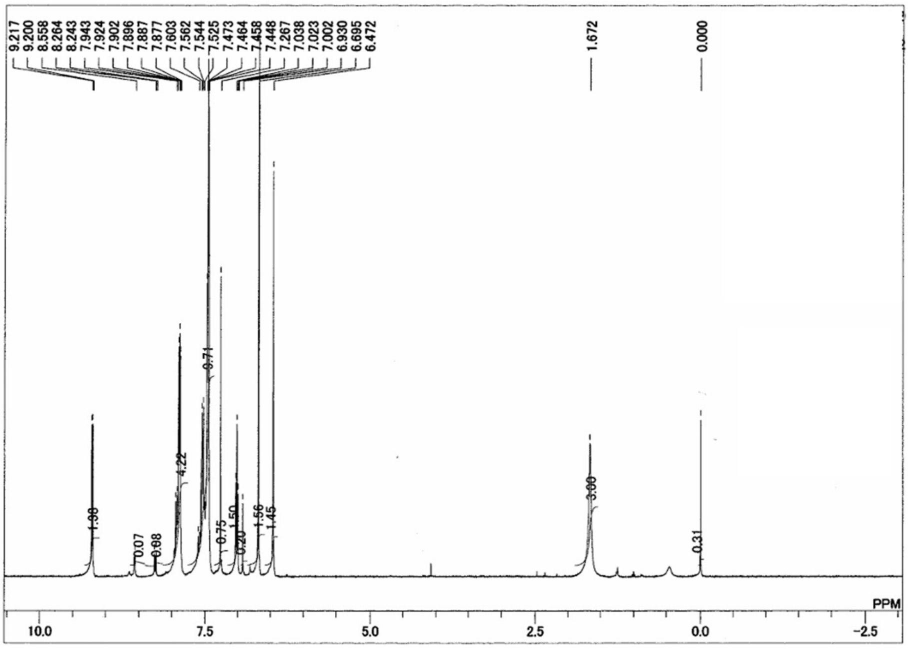

Figure S5. ^{1}H NMR spectrum (400 MHz, $CDCl_3$) of **5**.

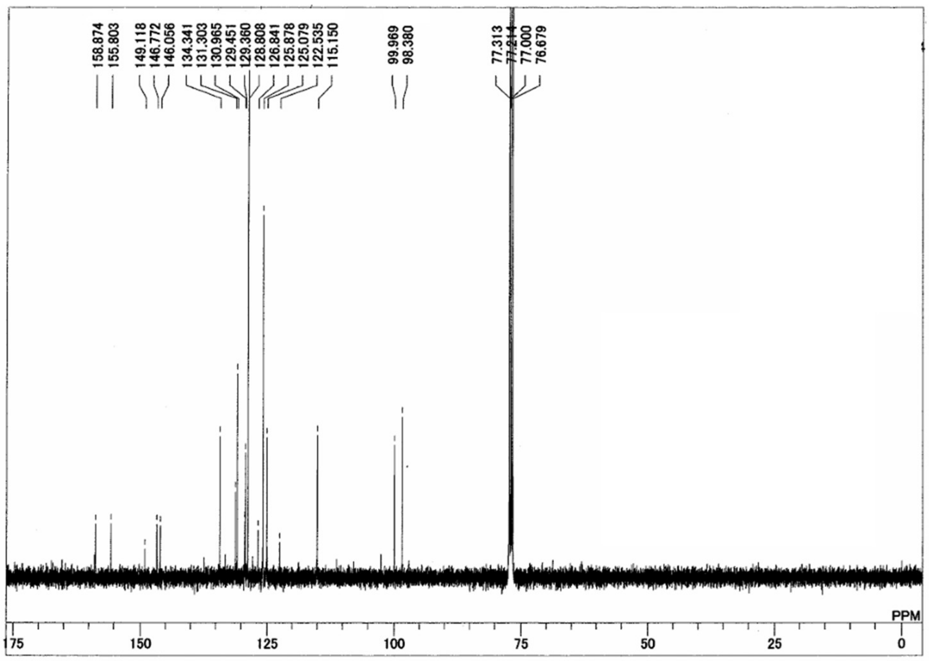

Figure S6. ^{13}C NMR spectrum (100 MHz, $CDCl_3$) of **5**.

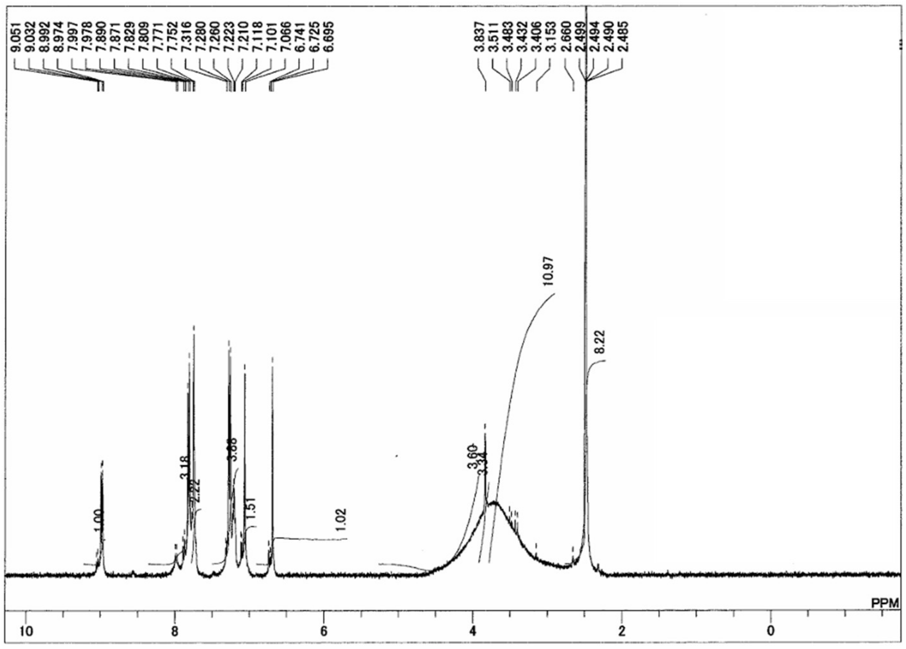

Figure S7. ^{1}H NMR spectrum (400 MHz, DMSO-d_6) of **6**.

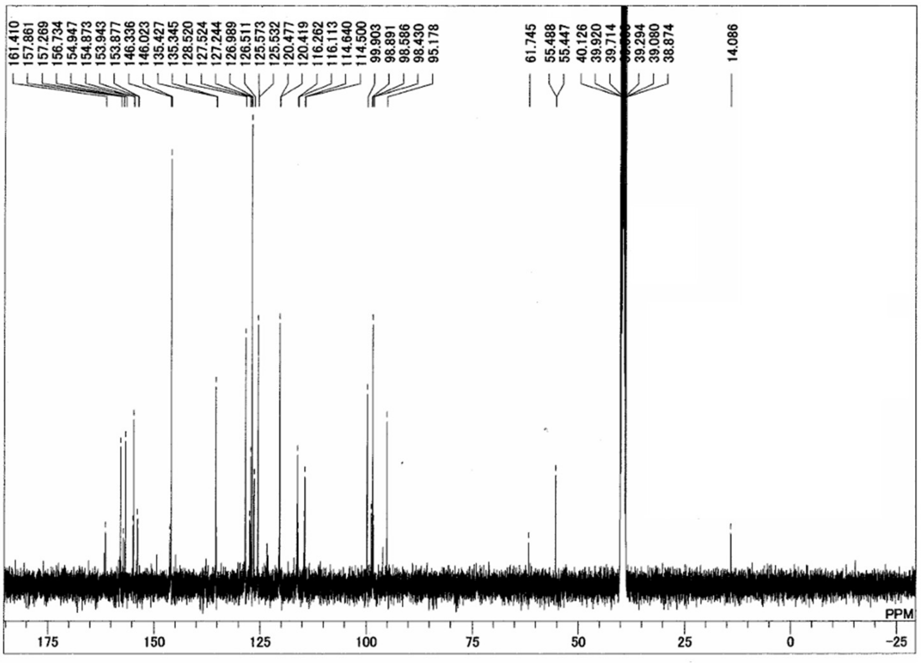

Figure S8. ^{13}C NMR spectrum (100 MHz, DMSO-d_6) of **6**.

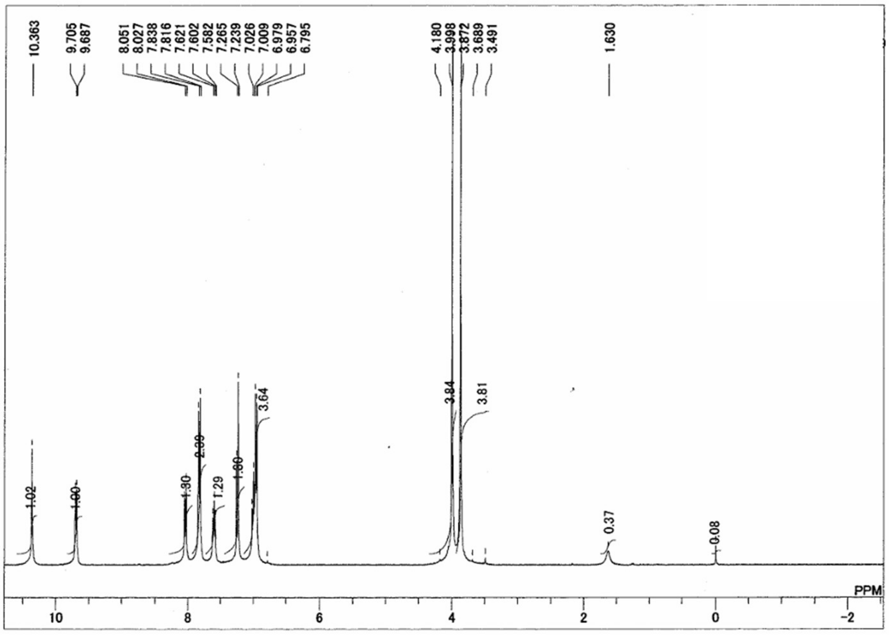

Figure S9. ^{1}H NMR spectrum (400 MHz, $CDCl_3$) of **7**.

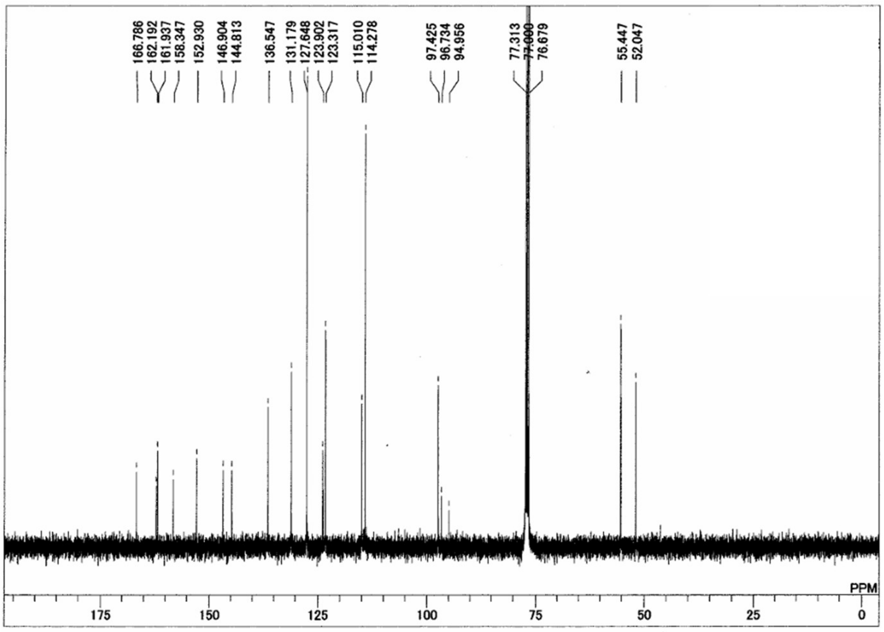

Figure S10. ^{13}C NMR spectrum (100 MHz, $CDCl_3$) of **7**.

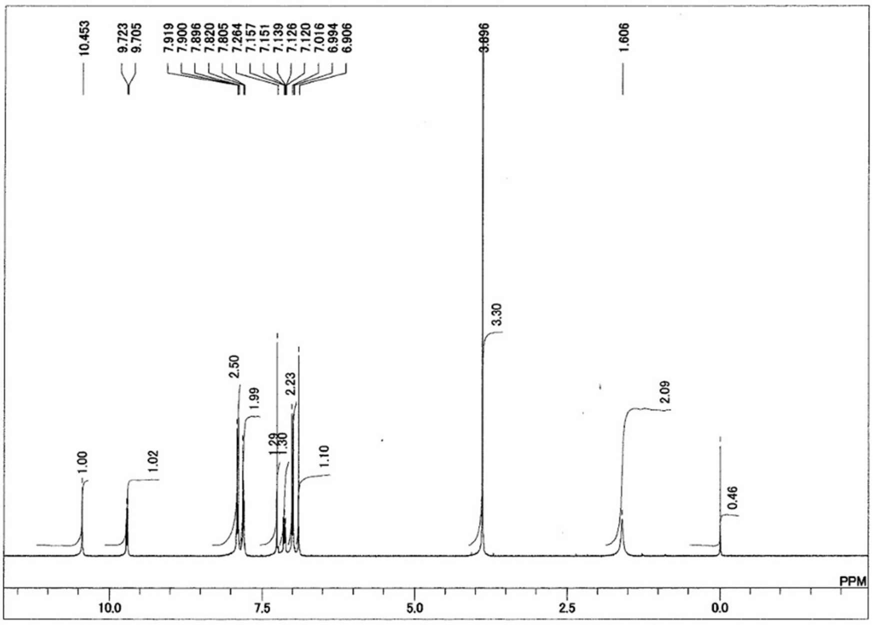

Figure S11. ^{1}H NMR spectrum (400 MHz, $CDCl_3$) of **8**.

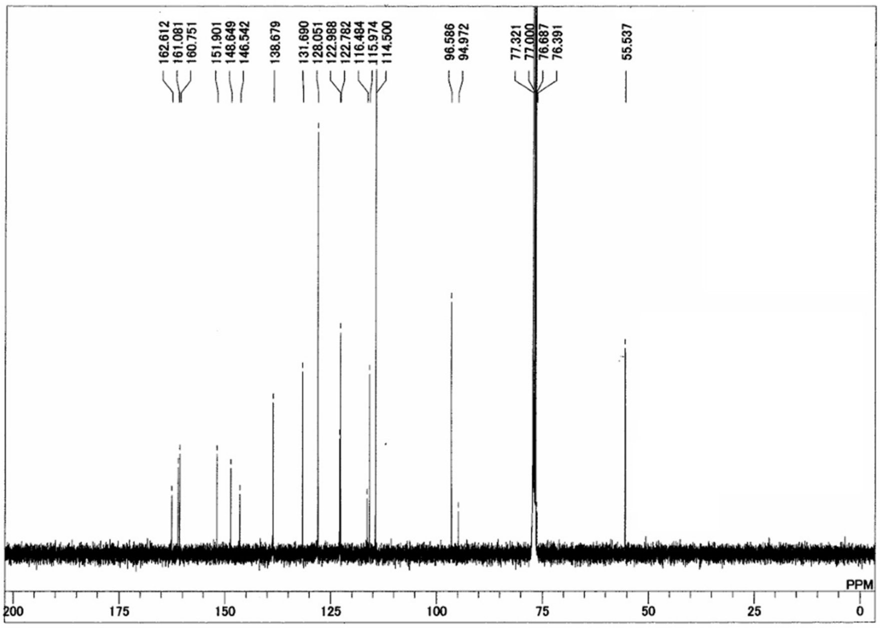

Figure S12. ^{13}C NMR spectrum (100 MHz, $CDCl_3$) of **8**.

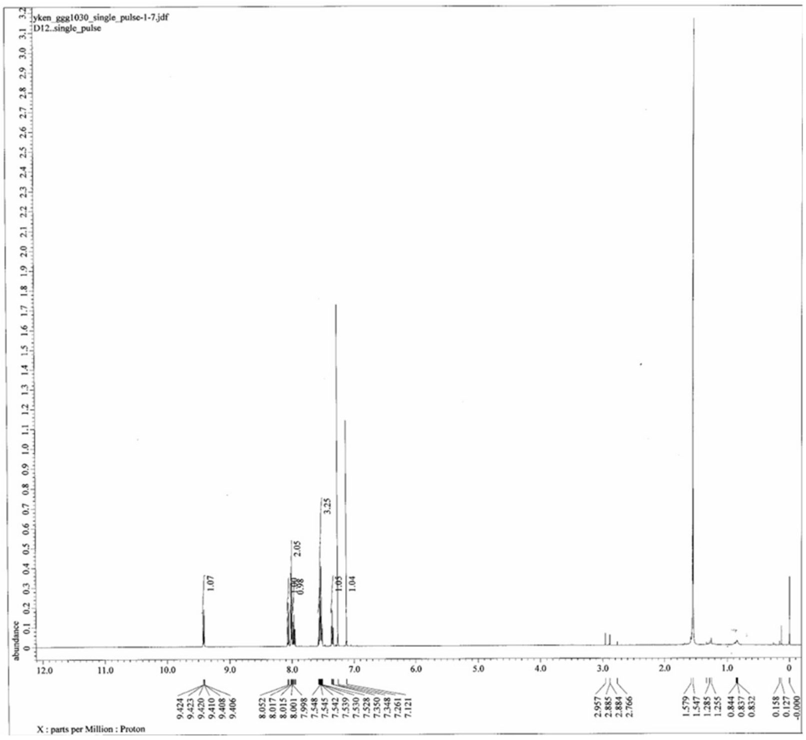

Figure S13. ^{1}H NMR spectrum (500 MHz, $CDCl_3$) of **9**.

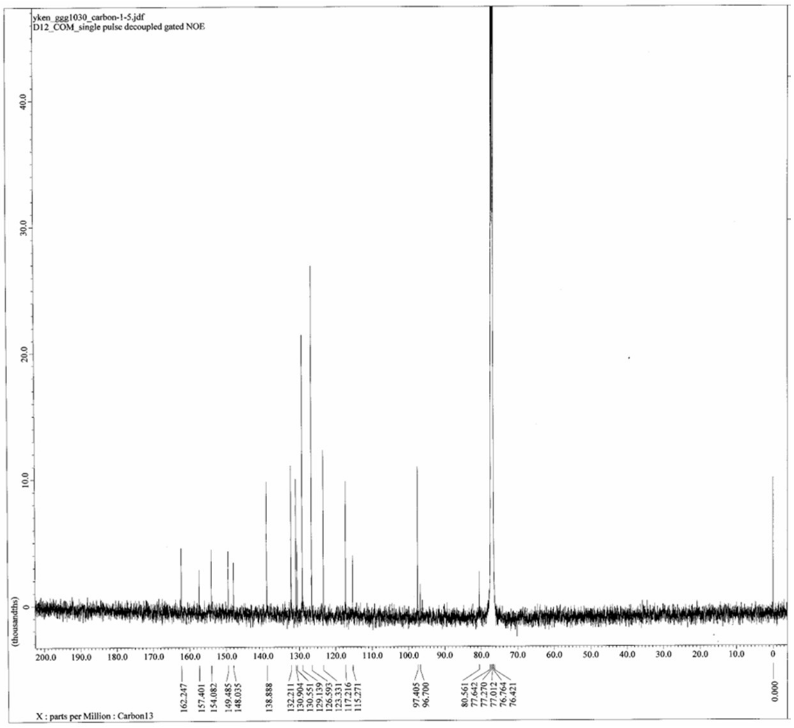

Figure S14. ^{13}C NMR spectrum (125 MHz, $CDCl_3$) of **9**.

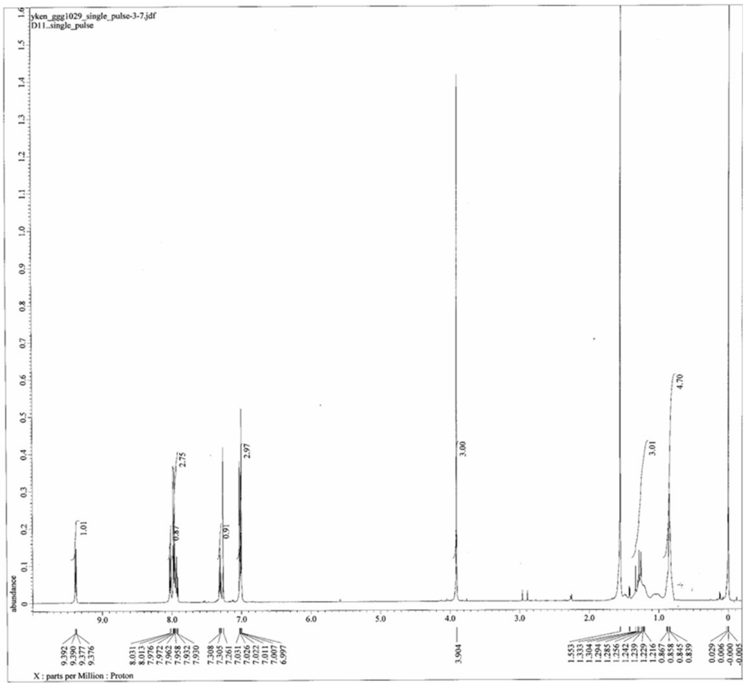

Figure S15. ^{1}H NMR spectrum (500 MHz, $CDCl_3$) of **10**.

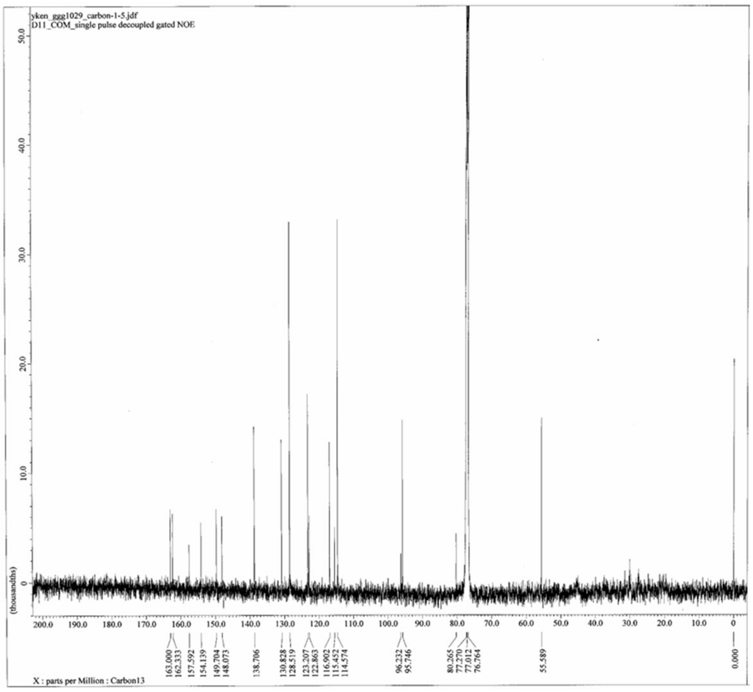

Figure S16. ^{13}C NMR spectrum (125 MHz, $CDCl_3$) of **10**.

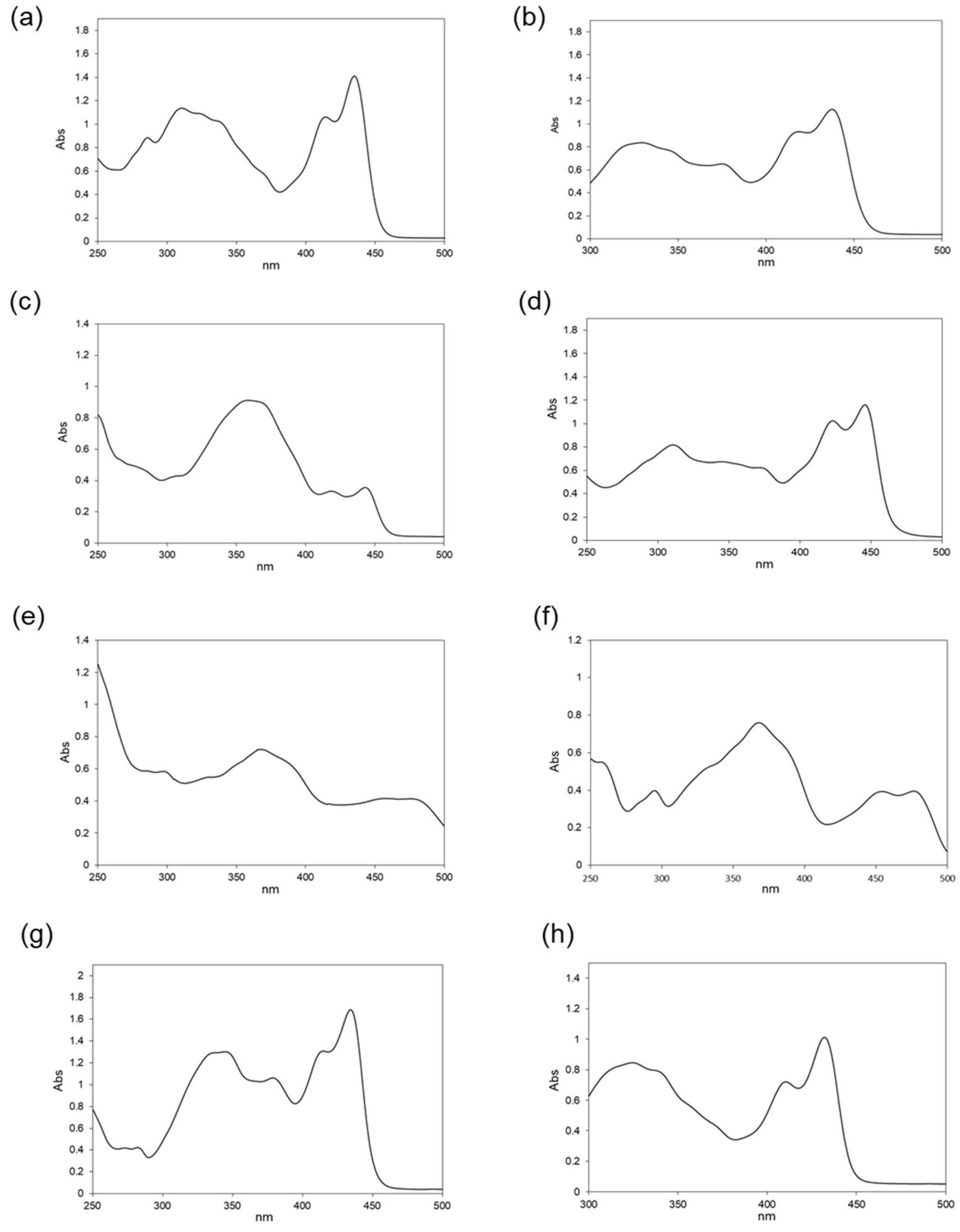

Figure S17. UV-Vis spectrum of **3-10** in EtOH.

(a) **3** (10^{-4} M); (b) **4** (10^{-4} M); (c) **5** (10^{-4} M); (d) **6** (10^{-4} M); (e) **7** (10^{-4} M); (f) **8** (10^{-4} M); (g) **9** (10^{-4} M); (h) **10** (10^{-4} M).

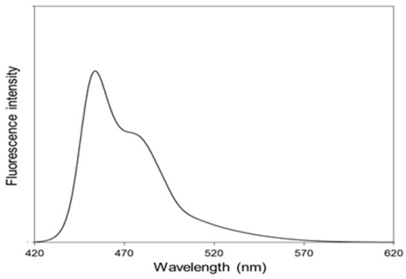

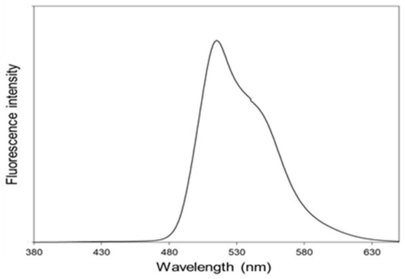

(c)

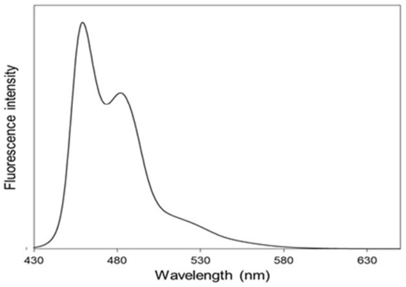

(d)

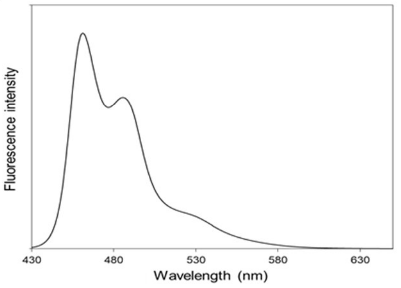

(e)

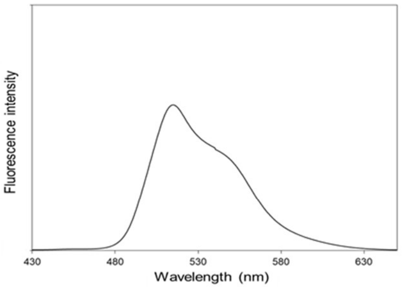

(f)

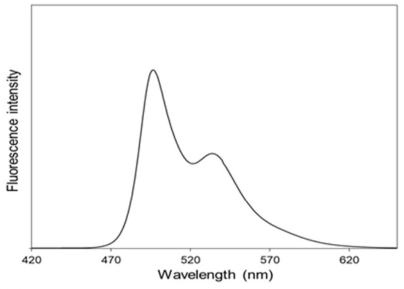

(g)

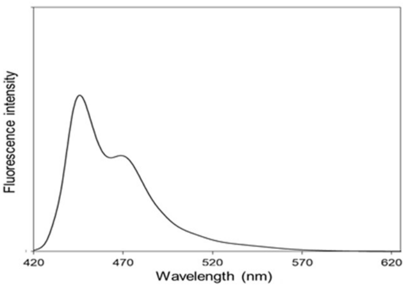

(h)

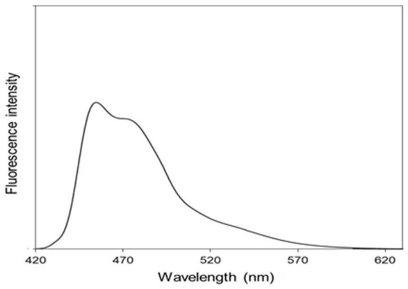

Figure S18. Fluorescence spectrum of **3-10** in EtOH.

(a) **3** (10^{-5} M, λ_{ex} = 435 nm); (b) **4** (10^{-5} M, λex = 438 nm); (c) **5** (10^{-5} M, λ_{ex} = 441 nm); (d) **6** (10^{-5} M, λ_{ex} = 445 nm); (e) **7** (10^{-5} M, λ_{ex} = 366 nm); (f) **8** (10^{-5} M, λ_{ex} = 368 nm)); (g) **9** (10^{-5} M, λ_{ex} = 432 nm); (h) **10** (10^{-5} M, λ_{ex} = 434 nm).

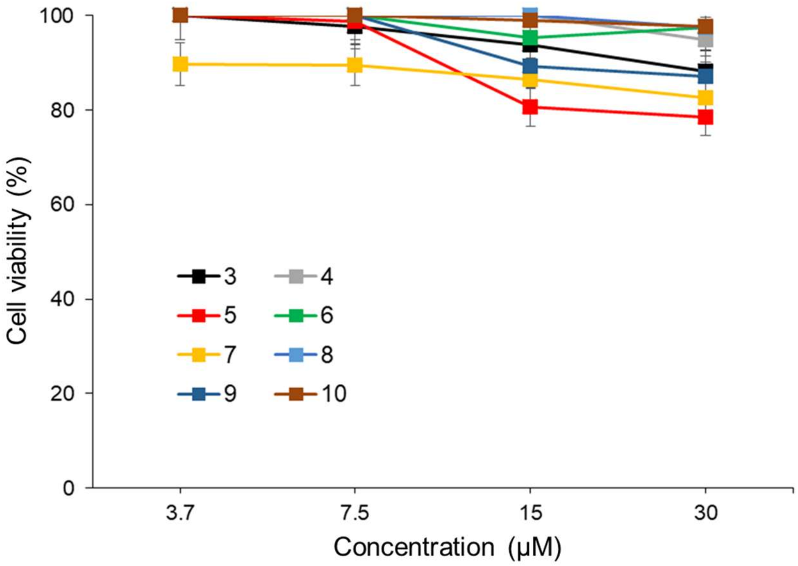

Figure S19. Anti-tumor activities of 3-phenyl pyrano[4,3-b]quinolizine compounds (**3-10**) against human colon cancer cells (Colo205) without 365 nm LED light irradiation.
